# Towards a broader view of the metabolome: untargeted profiling of soluble and bound polyphenols in plants

**DOI:** 10.1007/s00216-022-04134-z

**Published:** 2022-06-09

**Authors:** Maria Doppler, Christoph Bueschl, Florian Ertl, Jakob Woischitzschlaeger, Alexandra Parich, Rainer Schuhmacher

**Affiliations:** 1 Institute of Bioanalytics and Agro-Metabolomics, Department of Agrobiotechnology IFA-Tulln, University of Natural Resources and Life Sciences, Vienna, Konrad-Lorenz-Straße 20, 3430 Tulln, Austria; 2 Core Facility Bioactive Molecules: Screening and Analysis, University of Natural Resources and Life Sciences, Vienna, Konrad-Lorenz-Straße 20, 3430 Tulln, Austria

**Keywords:** Hydrolytic release, *Triticum aestivum*, Stable isotope, Tracer, Metabolomics, LC-HRMS

## Abstract

Phenylalanine (Phe) is a central precursor for numerous secondary plant metabolites with a multitude of biological functions. Recent studies on the fungal disease Fusarium head blight in wheat showed numerous Phe-derived defence metabolites to be induced in the presence of the pathogen. These studies also suggest a partial incorporation of Phe-derived secondary metabolites into the cell wall. To broaden the view of the metabolome to bound Phe derivatives, an existing approach using ^13^C-labelled Phe as tracer was extended. The developed workflow consists of three successive extractions with an acidified acetonitrile-methanol-water mixture to remove the soluble plant metabolites, followed by cell wall hydrolysis with 4M aqueous NaOH, acidification with aqueous HCl, and liquid-liquid extraction of the hydrolysate with ethyl acetate. The untargeted screening of Phe-derived metabolites revealed 156 soluble compounds and 90 compounds in the hydrolysed samples including known cell wall constituents like ferulic acid, coumaric acid, and tricin. Forty-nine metabolites were found exclusively in the hydrolysate. The average cumulative extraction yield of the soluble metabolites was 99.6%, with a range of 91.8 to 100%. Repeatability coefficients of variation of the protocol ranged from 10.5 to 25.9%, with a median of 16.3%. To demonstrate the suitability of the proposed method for a typical metabolomics application, mock-treated and *Fusarium graminearum*-treated wheat samples were compared. The study revealed differences between the hydrolysates of the two sample types, confirming the differential incorporation of Phe-derived metabolites into the cell wall under infection conditions.

**Supplementary Information:**

The online version contains supplementary material available at 10.1007/s00216-022-04134-z.

## Introduction

Polyphenols play important roles in defence and resistance against various types of biotic and abiotic stress, and are therefore interesting analytical targets for a large number of metabolomics studies. Most plant polyphenols are produced via the shikimate pathway through the aromatic amino acid phenylalanine (Phe). Phe derivatives can exhibit their biological function as antimicrobial or antioxidative metabolic end products directly, or constitute biosynthetic intermediates of other secondary metabolites. In addition, a number of phenolic compounds are incorporated into structural components like the cell wall.

For untargeted metabolomics approaches, aiming at the global extraction and measurement of soluble low molecular weight metabolites including polyphenols, sample preparation is usually limited to solvent extraction with mixtures of water and miscible organic solvents (e.g. methanol, ethanol or acetonitrile) and a few handling steps like dilution or centrifugation, while a sample clean-up is avoided to minimize discrimination of certain metabolite classes [1–3] or the formation of artefacts [4].

In contrast, cell wall-bound compounds cannot easily be extracted by solvents. Their analysis is further complicated by the complex structural network of different mono- and polymeric cell wall constituents. Plant cell walls consist of a network of cellulose fibres surrounded by a complex matrix of various polysaccharides, proteins, lignin and other phenolic compounds [5]. They can be roughly categorized into type I, (dicots and non-commelinoid monocots and gymnosperms) containing xyloglucans, pectins and structural proteins, and type II (commelinoid monocots), which are characterized by a large proportion of arabinoxylans and hydroxycinnamates with low levels of pectin and structural proteins [6, 7]. The cell walls of commelinoid monocots like grasses differ from dicot cell walls [7] primarily because xylans form their main hemicellulosic component, making up to 30% of the total cell wall [5]. Hydroxycinnamates like ferulic acid and coumaric acid are also present in significant quantities in the form of bound esters by linkage to xylans. Up to 4% esterified ferulic acid and up to 3% esterified coumaric acid were reported in the cell walls of grasses [7]. While coumaric acid is generally linked exclusively to polysaccharides, ferulic acid can also link two hemicellulose molecules, similar to structural proteins in dicots [7, 8], or form a bridge between hemicellulose and lignin via ester or ether linkage, respectively [7]. Interestingly, lignification of cell walls often starts from xylan-bound ferulate and coumarate moieties [5].

Approaches aiming to release the phenolic compounds incorporated into cell walls are mainly based on chemical or enzymatic treatment [9]. The hemicellulosic and lignin parts of the cell wall are of major interest with respect to polyphenols. The ester and ether bonds between polysaccharides, hydroxycinnamic acids, monolignols, lignans and flavonoids in the cell wall can be hydrolysed enzymatically, with aqueous acidic or alkaline solutions [10–14]. While acids are expected to cleave ether bonds, alkali mainly cleave ester bonds, liberating esterified hydroxycinnamates from cell wall polysaccharides [14]. Shahidi and Yeo [13] concluded in their review that the most common chemical method is alkaline hydrolysis at room temperature, as it has been proven to be suited for cleavage of ester bonds and at least partial cleavage of ether bonds; this method has also been recommended because, under alkaline conditions, the cell wall-released polyphenols have been described to be less prone to degradation compared to acidic conditions [13]. Monolignol moieties, like the coniferyl or hydroxyphenyl units of lignin, can also be released under alkaline conditions. In the presence of oxygen, β-O-4 bonds between monolignol units can be cleaved oxidatively to liberate vanillin or p-hydroxybenzaldehyde [15].

Plant cell walls are known to contribute significantly to plant defence as they are the first barrier that pathogens need to overcome for the successful invasion of plant cells. Pathogen attack and secretion of cell wall degrading enzymes stimulates several defence pathways, including cell wall reinforcement [6, 16]. Moreover, antimicrobial compounds can be stored in the cell wall and released during cell wall degradation [16]. In view of the great importance of the cell wall in the defence against pathogens and stress, it appears very interesting to extend metabolomics approaches to insoluble, bound polyphenols in order to investigate the process of cell wall fortification upon stress treatment or pathogen infection.

Here we present a protocol for the combined profiling of soluble and bound Phe-derived polyphenols in plants. The use of U-^13^C_9_ labelled Phe as a tracer allowed us to extend the range of Phe-derived metabolites to hydrolytically released polyphenols in an untargeted yet guided manner.

## Material and methods

### Chemicals

Methanol (MeOH) and acetonitrile (ACN) were obtained from Riedel de Haen, Honeywell (LC-MS grade, >99.9% purity, Seelze, Germany). Formic acid (MS grade), NaOH (>98% pure), HCl (MS grade), and ethyl acetate (>99.7% pure) were purchased from Sigma (Sigma Aldrich, Vienna, Austria). Water was purified by ion exchange and ultrafiltration with an ELGA system (PURELAB Ultra, Veolia Water, Vienna, Austria). ^13^C_9_-phenylalanine (99% isotopic purity, uniformly labelled) was purchased from Euriso-top (St Aubin, France). The following authentic reference standards were used for metabolite identification: phenylalanine (native, >99% pure, Roth), orientin (>99% pure, Extrasynthese), tricin (>95% pure, PhytoLab), ferulic acid (>99% pure, Fluka), vanillin (99% pure, Sigma-Aldrich), pinoresinol (95% pure, Sigma), chlorogenic acid (>95%, Sigma), and coumaric acid (>98%, Sigma). Coumaroylputrescine was kindly provided by G. Adam, BOKU.

### Cultivation and treatment of plants

Wheat plants of the genotype C4 [near isogenic line to Fusarium head blight (FHB)-susceptible Remus] were grown to the flowering stage in a greenhouse prior to treatment. Details about the individual steps of plant cultivation and the genetic background of the plants are presented in Doppler et al. [17] and Warth et al. [18].

At anthesis, flowering ears were treated with an aqueous U-^13^C_9_ Phe solution (5 g/l). For this, 10 μl aliquots were spiked to the left and the right floret of 10 consecutive spikelets, resulting in a total application of 200 μl (1 mg U-^13^C_9_ Phe, mock treatment) per wheatear. For the proof-of-concept study, another set of flowering wheatears was treated with either a *Fusarium graminearum* (Fg) spore solution (10^5^ spores/ml) or water (mock, control), as described in Doppler et al. [19]. Five biological replicates per experimental variant were harvested 96 h after treatment. The wheatears were cut above and below the highest and lowest treated spikelet, immediately frozen in liquid nitrogen, milled, and stored at −80°C until further analysis.

### Sample preparation

**Fig. 1:**
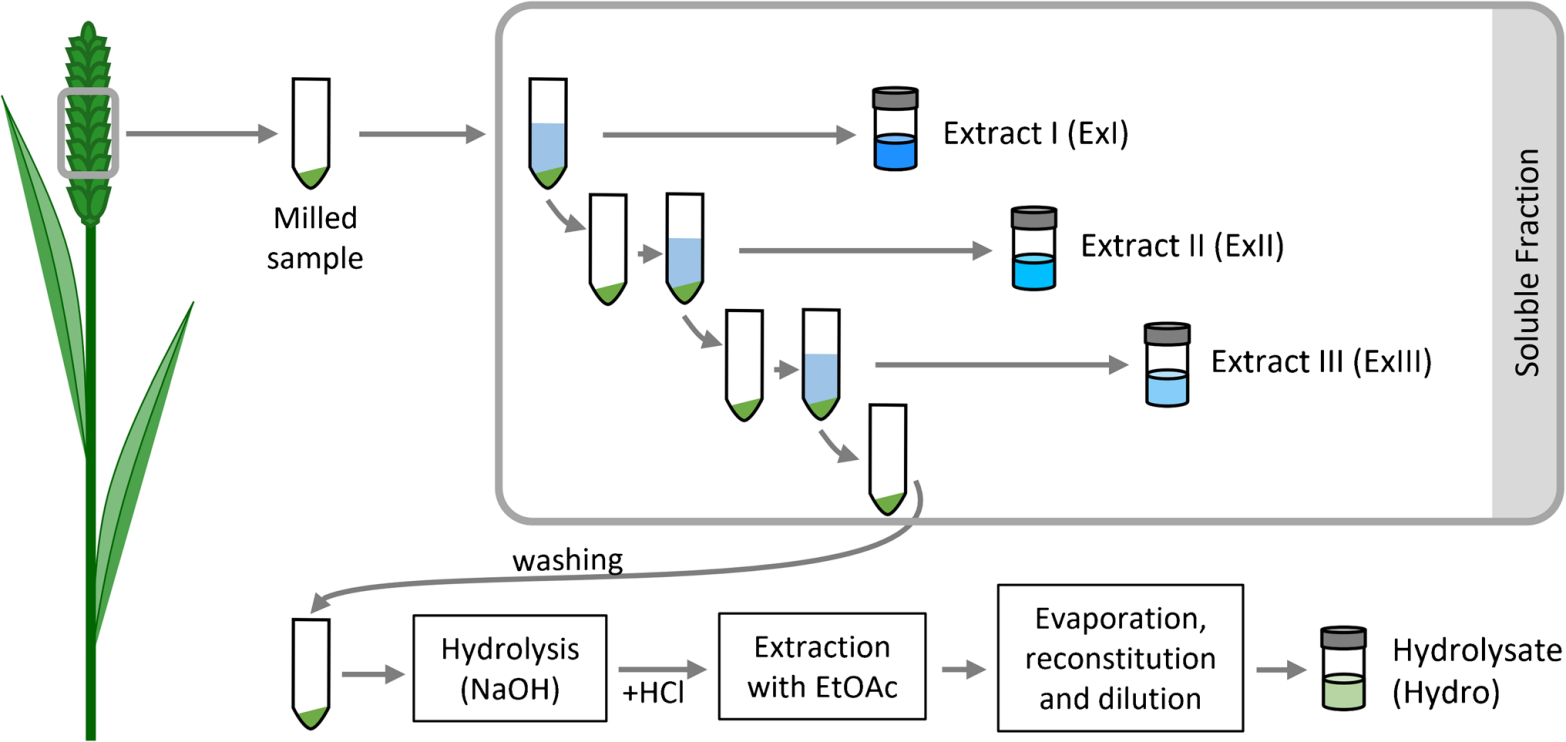
Overview of the sample preparation process and the derived sample types ExI, ExII, ExIII and Hydro

For each harvested wheat ear, four different sample types were generated. A schematic overview of the sample preparation process is depicted in Fig. 1. First, the soluble metabolites were extracted in three successive extractions resulting in extracts I, II and III (ExI, ExII, ExIII), respectively. The remaining pellet was washed and subsequently hydrolysed. The resulting hydrolysate was also prepared for measurement of the released low molecular weight constituents (Hydro).

### Extraction of soluble metabolites

Single extractions followed the optimized protocol described earlier by Doppler et al. [20]. In brief, the extraction was carried out with a mixture of water, acetonitrile and methanol [1:1.5:1.5 H_2_O:ACN:MeOH (v/v/v)] acidified with 0.1% formic acid (FA).

For this, 200 ± 2 mg of frozen, milled plant material was weighed and added to 2 ml of extraction solvent, then vortexed for 10 s and put into an ultrasonic bath for 15 min. After centrifugation [4°C; 1486 rcf (4500 rpm, *r*=65.5mm)] for 10 min, the supernatant was transferred to a new tube and the remaining pellet was used for the subsequent extraction. This extraction was repeated two more times, yielding extracts II and III. After extraction III, the remaining pellet was stored at 4°C until hydrolysis. All three extracts were treated separately, as follows: 600 μl was mixed with 300 μl H_2_O + 0.1% FA and centrifuged for 10 min [4°C; 4158 rcf (7500 rpm, r=66mm)]; 800 μl thereof was transferred to glass vials for liquid chromatography–high-resolution mass spectrometry (LC-HRMS) analysis.

### Cell wall hydrolysis

Hydrolytic release of polymer-bound cell wall constituents was based on the protocol of Bento-Silva et al. [10] with minor adaptions (Fig. 1): After the third extraction, the pellet was washed by adding 7 ml H_2_O. The mixture was gently mixed and centrifuged [6 min; 6°C; 6944 rcf (10,000 rpm, *r*=62mm)] and the supernatant was discarded. Hydrolysis was carried out by the addition of 3 ml 4M NaOH, gentle mixing, and shaking at room temperature for 18 h.

Next, 3.6 ml 3M HCl was added, the pH value was adjusted to pH 1.5 ± 0.1 and the sample was immediately extracted with 3 ml ethyl acetate (EtOAc) by vigorously shaking for 15 s. After centrifugation (10 min; 1621 rcf [4700 rpm, r=65.5mm]; 6°C) the EtOAc phase was transferred to a 16 ml glass vial. EtOAc extraction was repeated twice. The collected and combined EtOAc phases were dried using a speedvac system (Labconco CentriVap Cold Trap; 2.5 h; 10°C). The residue was reconstituted with 1 ml MeOH + 0.1% FA and 1 ml of H_2_O + 0.1% FA was added. After centrifugation [10 min; 6°C; 7392 rcf (10,000 rpm, *r*=66mm)], the clear sample solution was transferred to glass vials for LC-HRMS measurements.

### LC-HRMS measurements

For liquid chromatography, a Vanquish high-performance liquid chromatography (HPLC) system (Thermo Fisher Scientific, Bremen, Germany) was equipped with a C18 RP column (3.5 μm; 2.1 × 150 mm; XBridge^®^; Waters; Milford; MA; USA). Eluent A consisted of H_2_O + 0.1% FA (v/v) while eluent B consisted of MeOH + 0.1% FA (v/v). Gradient elution was carried out after injection of 2 μl sample aliquots with a constant flow rate of 0.25 ml/min and column temperature of 25°C, starting with 10% eluent B (hold time 1 min), increasing eluent B to 100% in 9 min. After a hold time of 3 min, the system was re-equilibrated for 7 min at 10% B, resulting in a total runtime of 20 min. The HPLC was coupled to a Q Exactive HF system (Thermo Fisher Scientific; Bremen; Germany) via a heated electrospray ionization (ESI) interface. Both positively and negatively charged ions were generated in fast polarity switching mode. Mass spectra were recorded in full scan mode with a resolution setting of 120,000 [full width at half maximum (FWHM) @*m/z* 200] and a mass range from *m/z* 100 to 1000. Mass spectra were recorded in the profile mode. For positive ionization, the spray voltage was set to 3500 V, and for the negative mode, to 3000 V. Sheath gas was set to 55 and aux gas to 5 (both arbitrary units).

The sequence was set up in a block design in randomized order including biological samples, solvent blanks, reference standards, and a quality control (QC) sample (QC standard mix), consisting of a mixture of standards in pure solvent. Measurement performance was monitored by regular injection and evaluation of a QC standard mix, consisting of 25 reference standards in MeOH/H_2_O/FA (1:1:0.01, v/v/v). The composition of the QC standard mix is provided in Online Resource 3.

### Data evaluation

After initial QC checks [verification of mass accuracy (<3 ppm), retention time stability (<4 s), peak areas for technical replicates of the QC standard mix (<10% relative standard variation)], data evaluation was carried out in an untargeted manner with the TracExtract module of the MetExtract II software package [21]. Briefly summarized, this tool searched for co-eluting pairs of native and partly ^13^C-labelled Phe-derived biotransformation products and constructed a data matrix containing all detected native and partly ^13^C-labelled biotransformation products of the Phe tracer. Additionally, MetExtract II was used to search for native monoisotopic and ^13^Cx-labelled isotopologues in any sample where the respective chromatographic peaks had not been detected (including samples not having been spiked with the ^13^C-Phe tracer) in a targeted manner and integrated their peak areas. Parameter settings for TracExtract were as follows: ^13^C-Phe spiked samples: used for data matrix generation and metabolite convolution, any other samples not having been spiked with the ^13^C-Phe: omit features detected in these samples as false positives, minimum signal intensity: 1000, ^13^C-enrichment in the ^13^C_9_ tracer: 99.5%, maximum allowed mass deviation: 3 ppm, chromatographic peak width: 3–19 scans, minimum chromatographic peak shape similarity for feature grouping (Pearson correlation): 0.85. All parameters for data evaluation with MetExtract II are listed in Online Resource 1. Finally, all detected biotransformation products of Phe were manually verified with the vendor software Xcalibur (Thermo Fisher). Any putative biotransformation product that was a false positive (e.g. noise incorrectly assumed to be a chromatographic peak, detected in the blanks) was removed from the data matrix during this manual step. Metabolite identification according to level 1 was carried out by comparison with authentic reference standards as described by Blaženović et al. [22]. For this, *m/z*, ionization behaviour, retention times and MS/MS fragment spectra were manually compared.

### Statistical analysis

Statistical analysis was carried out with a customized R script (R version 3.5.9 [23]). The data matrix generated by MetExtract II was used after manual curation. If not otherwise specified, the peak areas of the native, monoisotopic isotopologues of the detected biotransformation products were used.

Venn diagrams: A metabolite was counted as present in a particular experimental variant if it was detected as the native metabolite form in at least four out of five replicates of the particular group. Otherwise, it was not considered for this group. Heatmaps: Data were auto-scaled (mean centring, standard deviation scaling), and missing values were imputed with 0 [24]. The heatmap illustrations were generated with the gplots R package (version 3.0.1.1; https://www.rdocumentation.org/packages/gplots/versions/3.1.1) using squared Euclidean distance and Ward’s linkage method.

PCA: For principal component analysis (PCA), the data matrix was auto-scaled, and missing values were imputed with 0. The scores and loadings plots were illustrated with the ggbiplot R package (version 0.55; https://www.rdocumentation.org/packages/ggbiplot/versions/0.55). RSD plots: To illustrate the intra-group variance in peak areas within the experimental groups, the relative standard deviation (RSD) was calculated for each sample type (ExI, ExII, ExIII, Hydro, respectively) and each metabolite separately and illustrated in the form of a histogram.

To assess the completeness of the three consecutive extractions, two method performance parameters were calculated: (a) single extraction efficiency (SEE) (i.e. the relative amount of metabolite *x* extracted with a single extraction, in %) and (b) cumulative extraction yield (CEY) after three consecutive extractions (i.e. the total yield of metabolite *x* that was achieved with three consecutive extractions, in %). The SEE of a metabolite was set to 100% for metabolites detected only in the first extract and not in the second or third extract, or (ii) [1 – amount(extraction II)/amount(extraction I)]*100 in % for metabolites detected in the first and second extract (or also the third extract). The CEY of a metabolite was calculated with the formula *y* + (1 – *y*) * *y* + ( 1 – *y* – (1 – *y*) * *y*) * *y*, with *y* being the SEE of the particular metabolite. The calculations were carried out with the averaged (average value of replicate extractions) and range-scaled (maximum value set to 1) peak areas of the respective metabolite ions.

## Results and discussion

**Fig. 2:**
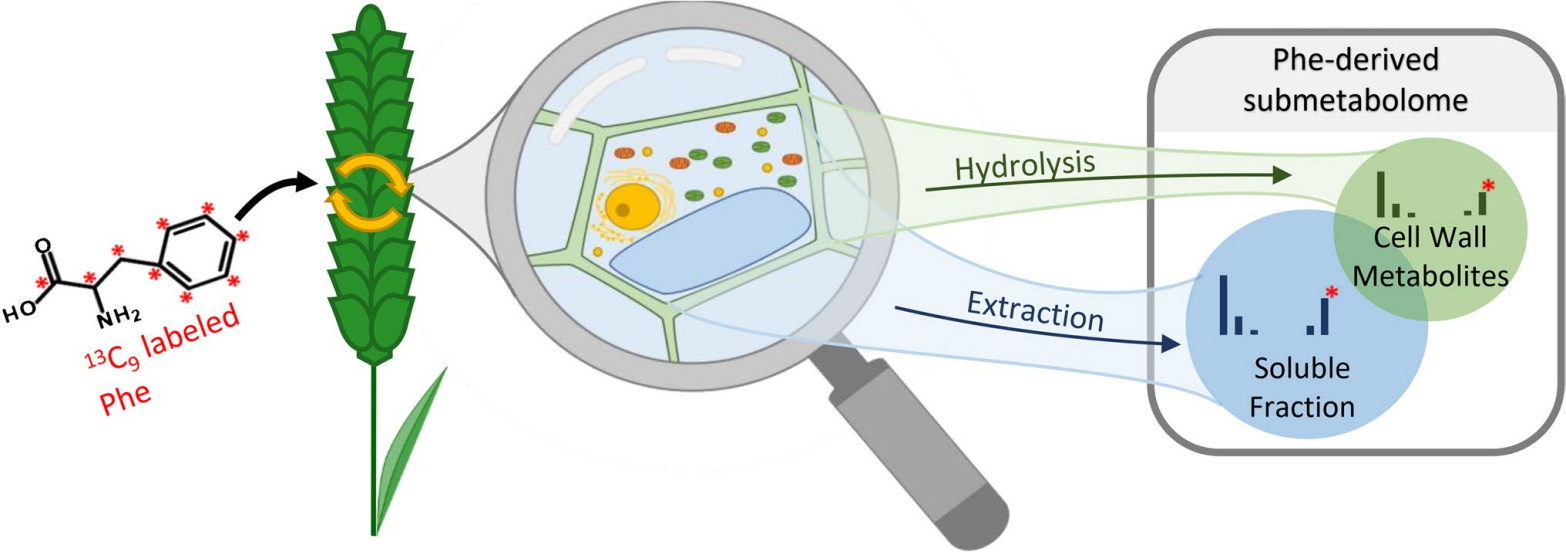
Graphical illustration of the principle of isotope-assisted screening of soluble and cell-wall bound Phe-derived polyphenols in this work

Raw LC-HRMS data were first inspected to verify consistency and measurement performance by testing the acquired QC samples for mass accuracy, peak intensity, and retention time stability. This test was passed for all 25 compounds of the QC mix. Subsequently, data were evaluated with MetExtract II [21] to detect all metabolites, derived from transformation of the U-^13^C_9_-Phe tracer. This software screens the raw LC-HRMS data for characteristic isotope patterns of biotransformation products derived from both the endogenous native and the exogenously applied ^13^C-labelled Phe (Fig. 2). Thereby, it not only selectively and efficiently detects biotransformation products of interest (i.e. a low rate of false positives detected in blank samples and wheatear samples not treated with the ^13^C-labelled Phe tracer was reported), but it also allows the determination of the number of carbon atoms contributed by the Phe tracer to any biotransformation product (e.g. 7 for vanillin). Moreover, compounds containing two or more Phe tracer units (e.g. lignans) can be searched for. After manual removal of the 16 false positives, which accounted for 2.6% of all detected feature pairs, the remaining putative descendants of Phe were compiled into a data matrix (Online Resource 2). As true ^13^C-Phe-derived polyphenols can only be detected in ^13^C-Phe-treated samples, the so detected polyphenol ions were used as targets and searched for in the Fg-treated samples of the proof-of-concept study which had not been treated with the ^13^C tracer. This enabled the semi-targeted evaluation of all experimental mock- and Fg- treated wheat samples without the need for labelling with the expensive ^13^C-Phe. In addition, any potential alteration of the metabolic state of the experimental samples due to treatment with the tracer can be avoided.

For the detailed characterization and validation of the presented approach, only the mock-treated wheat samples were used. After raw data processing, the detected polyphenol compounds were subjected to statistical analysis, using the ^12^C peak areas of the most abundant metabolic feature per metabolite.

### Number and complementarity of detected metabolites

In total, 624 metabolite ions (i.e. features), corresponding to 209 Phe-derived metabolites, were detected in the extracts (ExI-ExIII) of the mock-treated wheat samples and/or the alkaline hydrolysate of the remaining cell pellet. Of these 209 metabolites, 62 were detected in both ionization modes, while 27 compounds were solely detected in the positive mode and another 120 in the negative mode only.

Four of the 209 metabolites were excluded from further statistical analysis, as they did not fulfill the criterion to be present in at least 4 of 5 biological replicates. This resulted in a final set of 205 metabolites, 156 of which were detected in either one, two or all three extracts (ExI, ExII, ExIII) and thus represent the soluble fraction. In addition, 49 metabolites were solely detected in the hydrolysates and therefore represent bound and hydrolytically released metabolites.

**Fig. 3:**
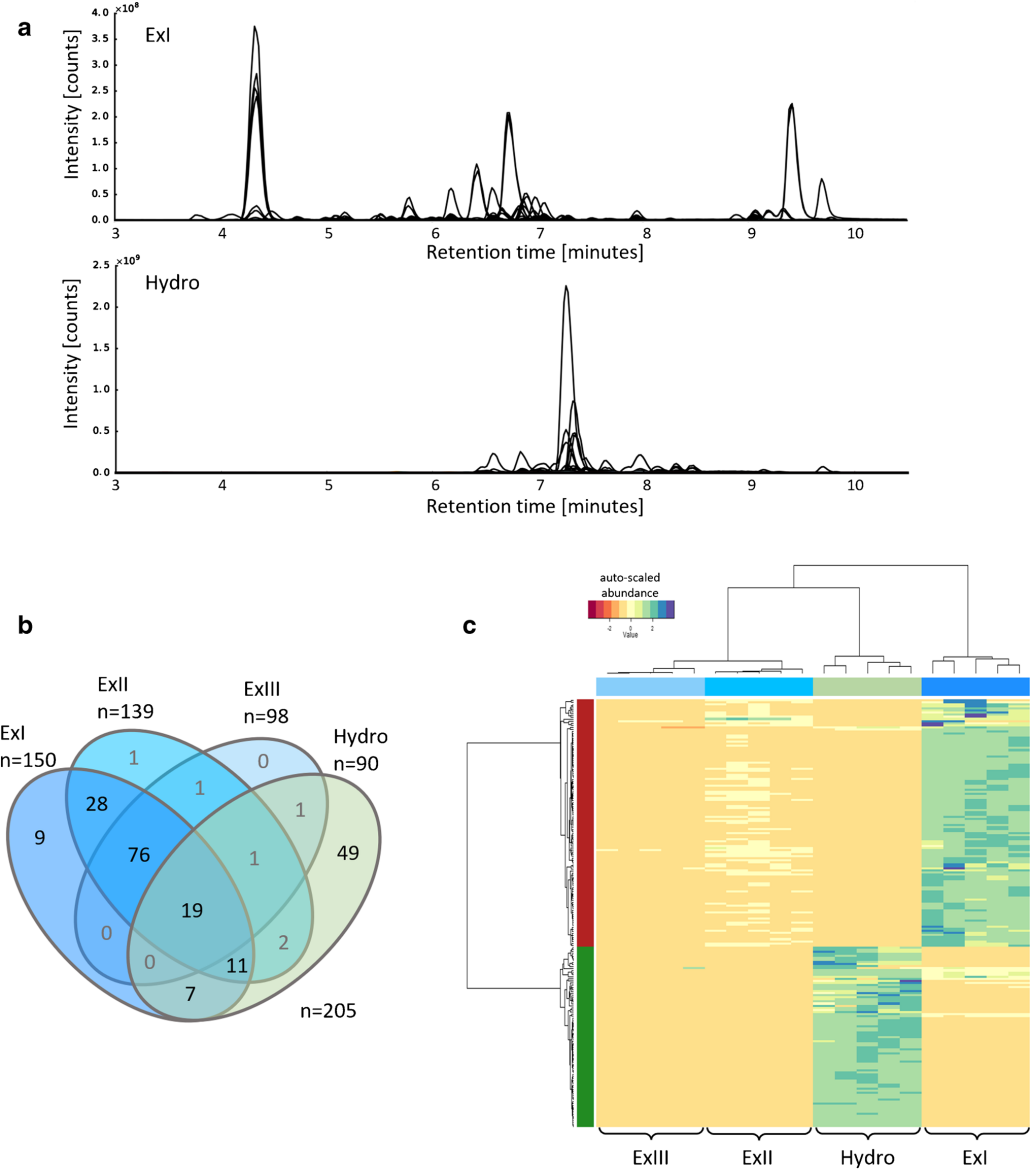
**a** Overlay of extracted ion chromatograms of metabolites detected in extract I (ExI) and hydrolysate (Hydro). Each peak represents a MetExtract II derived monoisotopic ^12^C EIC trace. **b** Venn diagram of the detected metabolites (metabolites need to be present in at least 4/5 replicates to be depicted; 4 metabolites did not fulfil these criteria). **c** Heatmap of all detected metabolites and all four sample types, the two main metabolite clusters are indicated with red and green bars on the left side

Clear differences between the LC-HRMS metabolite profiles of extracts and hydrolysate samples were observed. While the soluble metabolites (exemplified with ExI samples) eluted from the HPLC column mainly between 4 and 10 min, metabolites released from the cell wall by alkaline hydrolysis eluted in a narrower retention time window between 6.5 and 9 min (Fig. 3a).

The distribution of the 205 metabolites, consistently detected in ≥4 of 5 replicates over the different sample types, is shown in Fig. 3b. As expected, the ExI samples contained the highest number of metabolites (*n*=150), followed by ExII with *n*=139 and ExIII with *n*=98. Among the 90 metabolites detected in the hydrolysate samples, 41 were also detected in the soluble fraction, while 49 Phe derivatives were exclusively present in the Hydro samples. These results demonstrate that the combination of solvent extraction with alkaline hydrolysis provides complementary information on extractable polyphenols, potentially acting as metabolic precursors for stress-mediated cell wall fortification. Moreover, the Phe-derived compounds that are only accessible after cleavage from the cell polymer matrix can provide additional insight into the composition of the cell wall under different experimental conditions. This complementarity can also be seen in the heatmap after hierarchical cluster analysis (Fig. 3c). The extracts and hydrolysate (top dendrogram) clearly cluster according to sample type (Ex I-III and Hydro), while the metabolite ions (left dendrogram) roughly group into soluble compounds (red cluster) and those bound to the cell wall, which were exclusively present or more abundant in the hydrolysate samples (green cluster). Overall, 156 soluble Phe derivatives were detected in extracts ExI, ExII and ExIII. Among those, 16 (9 plus 7) metabolites were extracted quantitatively with a single extraction and were therefore not found in the second or third extract. Another 39 (28 plus 11) of the 150 metabolites found in ExI were also detected in ExII but not in ExIII, indicating their complete extraction after two successive extractions with the applied method. Ninety-five of the remaining (76 plus 19) soluble metabolites were detected in each of the three successively prepared extracts. Therefore, for these compounds, incomplete extraction before hydrolysis has to be assumed. In addition, a few metabolites were detected only in ExII, ExIII or in combination with Hydro. They were inspected manually and classified as data processing artefacts caused by low abundant signals or noise and were therefore excluded from further statistical analysis.

### Extraction efficiency and precision of the method

With each of the consecutive solid-liquid extractions, a proportion of the soluble metabolites is transferred into the liquid extract. The extraction efficiency per single extraction (SEE) can be assumed to be constant for successive extractions, but may differ between metabolites. Consequently, assuming a high SEE value, several consecutive extractions remove a large proportion of the soluble metabolites from the sample. The cumulative recovery of *x* consecutive extractions can be calculated from SEE and is termed cumulative extraction yield (CEY).

**Fig. 4:**
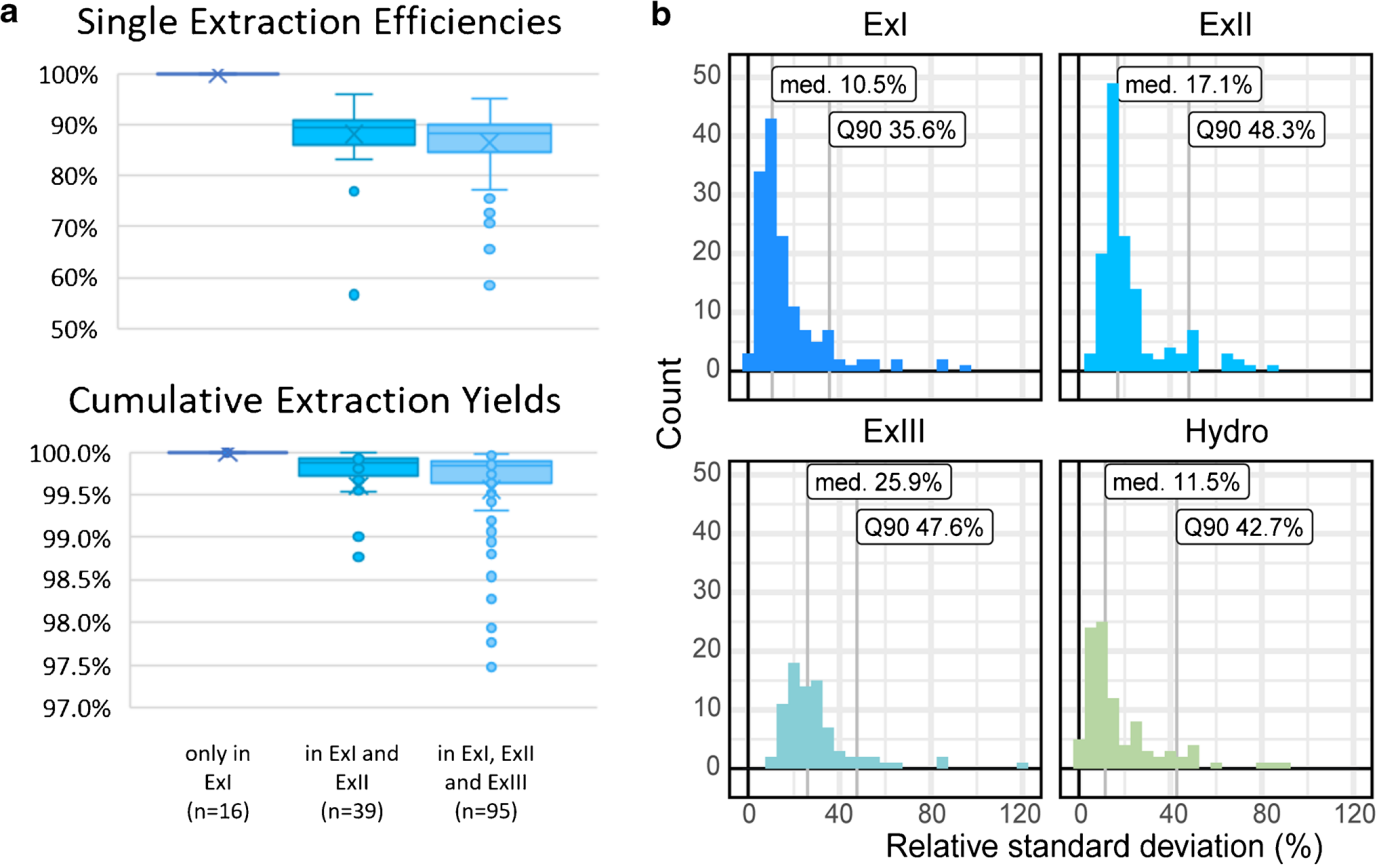
**a** Single extraction efficiencies (SSEs) and cumulative extraction yields (CEYs) for metabolites detected (i) only in ExI, (ii) in ExI and ExII, and (iii) in ExI, ExII and ExIII **b** Distribution of relative standard deviations (median and Q90) of peak areas for all metabolites and sample types

Based on the average peak area ratios obtained for the successive extractions, SEEs were calculated for those 150 soluble metabolites consistently detected in the replicates of ExI. For metabolites detected only in ExI and not in any subsequent extract (ExII or ExIII), the SEE can be assumed to be practically 100%. Contrarily, for any metabolite also detected after the second (ExII) and/or third (ExIII) extraction, the SEE was calculated from the averaged values of the metabolite abundance in the samples of the second and first extracts. SEEs were estimated separately for each of the three different cases (detected only in ExI; detected in ExI and ExII; detected in ExI, ExII and ExIII) and are depicted in Fig. 4a. We assumed SEEs of 100% for the 16 metabolites detected only in the first extract (ExI) (Venn diagram, Fig. 3b). SEEs of the 39 metabolites detected in ExI and ExII (but not in ExIII) were on average at 88.1%, with a standard deviation of 6.6%, while for the 95 metabolites detected in all three extracts, an average SEE of 86.5% with a standard deviation of 6.0% was obtained. Furthermore, cumulative extraction yields (CEYs) achieved after three consecutive extractions were also calculated for the 150 soluble metabolites (Fig. 4a). For each of the three categories (ExI only; ExI and ExII; EXI, ExII, ExIII), the averaged CEY values ranged above 99.5% These results demonstrate that with three successive extractions, more than 99% of the soluble metabolites were extracted from the sample. Therefore, any phenylalanine derivative detected in the alkaline hydrolysate after three successive extractions can be considered to represent a bound, insoluble metabolite which was truly released from the polymeric cell matrix. Moreover, the CEY values being close to 100% and the consistent high SEE values obtained from extraction to extraction indicate that for the large majority of the detected Phe derivatives, inaccurate comparative metabolite quantification due to signal suppression/signal enhancement in the ESI source can be neglected under the applied conditions. For SEE values of individual metabolites, see online resource 2.

Additionally, it should be mentioned that, depending on the analytes under investigation and the purpose of the study, a single extraction may be sufficient before hydrolysis, for example if no clear differentiation between soluble and bound metabolites is required.

As a measure of the repeatability of the method, individual relative standard deviations (RSDs) of the peak areas (*n*=5 replicates) were calculated for each metabolite and sample type. Their distribution is depicted in the form of histograms together with the median and 90th quantile RSDs (Fig. 4b). The median RSD ranged from 10.5 to 25.9% for all sample types, while 90% of all detected metabolites obtained lower variability than 35.6 to 48.3%. The lower precision for ExII and ExIII compared to the ExI and Hydro samples may arise from the generally lower metabolite abundance as well as from the additional steps of repeated extraction during sample preparation.

### Targeted verification of metabolites

**Fig. 5:**
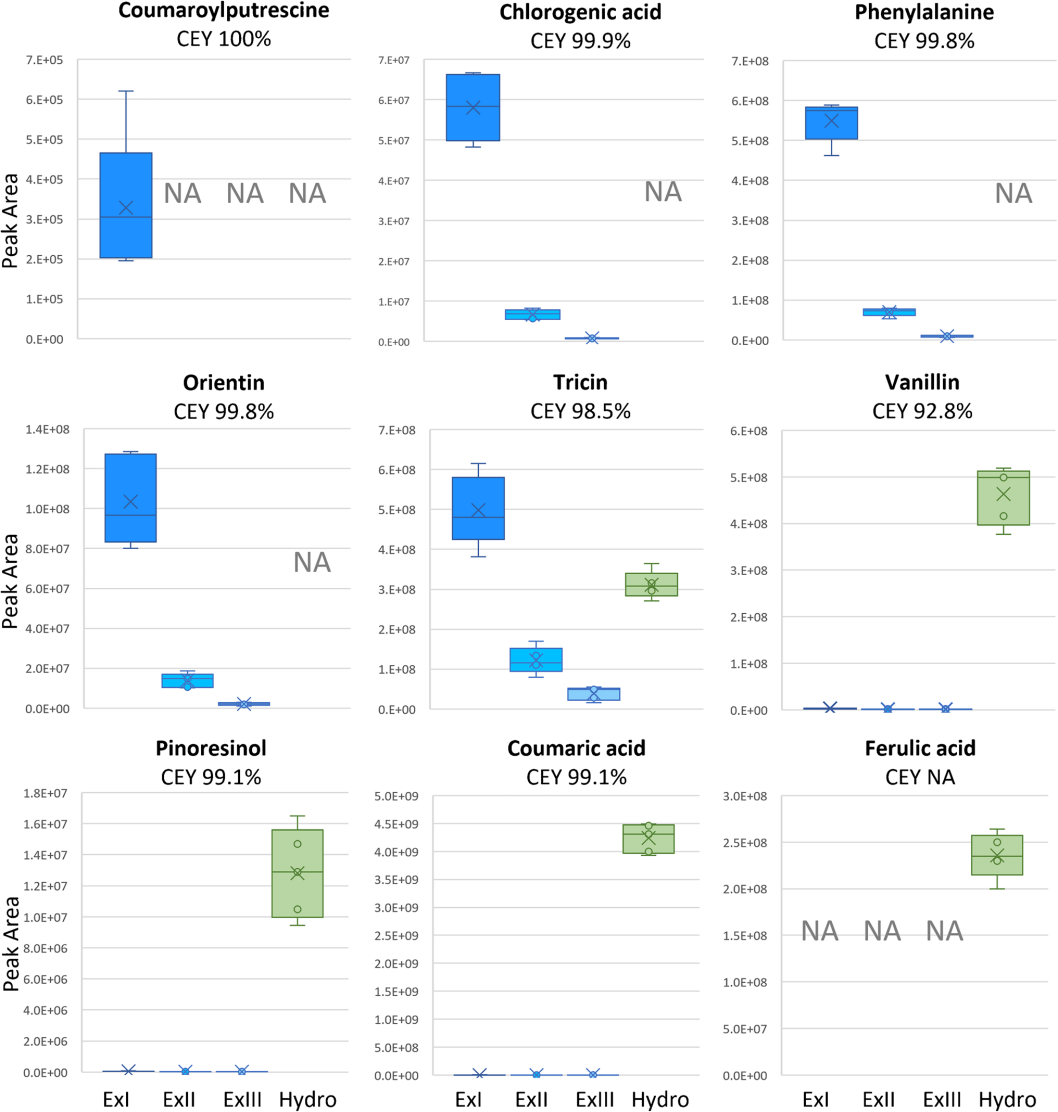
Box plots of peak areas and total (i.e. cumulative) extraction yields of nine identified wheat metabolites in ExI, ExII, ExIII and Hydro samples. NA indicates that the particular metabolite was not detected in the respective sample type. CEY: cumulative extraction yield of the three consecutive liquid extractions

Of the 209 Phe-derived metabolites found either in the liquid extracts or after hydrolysis and liquid/liquid extraction, the identity of 16 metabolites was successfully confirmed by comparison with authentic reference standards [22]. As an additional plausibility check and to illustrate typical metabolite distributions, for nine of those, the relative abundance was plotted and manually inspected across the four sample types (Fig. 5).

Coumaroylputrescine (only detected in ExI), chlorogenic acid, Phe and orientin (detected in ExI, ExII, and ExIII), represent four metabolites that were assigned to the soluble fractions only. While coumaroylputrescine and chlorogenic acid were exclusively detected in ExI and ExI, ExII, and ExIII, respectively, very small amounts of Phe and orientin had originally also been found in the hydrolysates (data not shown), but due to their rather low abundance ratio of Hydro to ExIII, these two metabolites were removed from the list of hydrolysate compounds (i.e. they are not illustrated in Fig. 5), since a carryover from the soluble into the Hydro samples could not be 100% excluded. This is also in agreement with current knowledge; these two compounds have so far not been described to be bound to the insoluble cell wall polymers of grasses like wheat. In contrast, tricin, vanillin, pinoresinol, and coumaric acid were present in all four sample types. Flavonoids are a diverse group of polyphenols deriving from Phe with a wide range of biological functions [17, 25]. Interestingly, both orientin and tricin are already known to be involved in the plant defence response to biotic and abiotic stress [26, 27]. While orientin can be attributed to the soluble fraction (Fig. 5), tricin has been described to be covalently bound to lignin via a 4′-O- ether bond. Tricin was suggested to act as nucleation site of lignification and should therefore be localized at the initiating end of its lignin moiety [28, 29]. In agreement with this, tricin was detected in the extract samples, but when compared with ExIII, a greater abundance of signals was detected in the hydrolysate. This indicates that tricin is both a soluble wheat metabolite, and incorporated in the cell wall and released by hydrolysis. In contrast, vanillin, pinoresinol, coumaric acid and ferulic acid were mainly detected in the hydrolysate samples. While vanillin, pinoresinol and coumaric acid were still found at low levels in the extracts, ferulic acid was only found in the hydrolysate. Vanillin is a known product of lignin hydrolysis [30]. Under alkaline conditions in the presence of oxygen, β-O-4 bound coniferyl units can be oxidatively cleaved to vanillin, which can then be found as soluble constituent in the hydrolysate [15]. This behaviour is also reflected in our study, in which 4M NaOH was used for hydrolysis. Pinoresinol consists of two coniferyl alcohol (i.e. monolignol) units that are conjugated via radical coupling and randomly polymerized into lignin or alternatively conjugated to other cell polymer matrix components like carbohydrates [14, 31]. Consistent with this, the majority of pinoresinol was found in the Hydro samples. Additional studies will be necessary to clarify whether pinoresinol is mainly released as an optically inactive lignan from randomly formed lignin or alternatively cleaved as a specific optical isomer from other cell wall components as has been reported for enzymatically hydrolysed whole grain cereal samples [31]. Coumaric acid and ferulic acid are hydroxycinnamic acids (HCAs) known to be prominently abundant in the cell wall of grasses, including wheat [5, 7]. Ferulic acid is the most abundant HCA in wheat [32] and is known to be covalently linked to hemicellulose polysaccharides via ester bonds and lignin via ether bonds, whereas coumaric acid has mainly been reported to bind to hydroxyl groups of polysaccharides by ester formation in various ways [7, 33, 34]. Interestingly, among the highly abundant metabolites in the hydrolysate samples, many were annotated as di- or trimers of ferulic acid, indicating the different ways ferulic acid is incorporated in the cell wall and released by the presented protocol [7, 8].

### Proof of concept: application to an untargeted metabolomics study

**Fig. 6:**
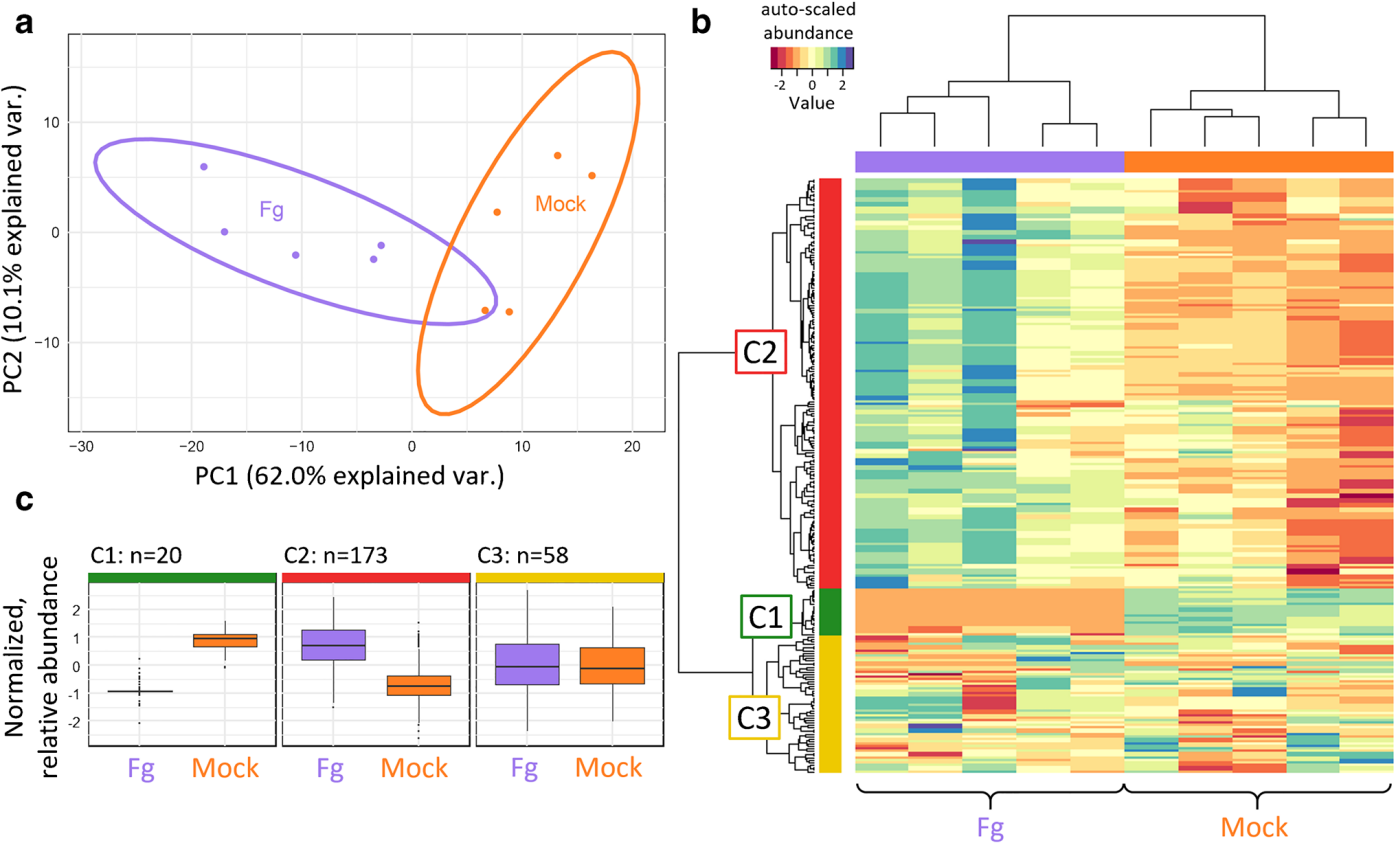
Proof-of-concept study. **a** PCA for the two sample types (mock and Fg treatment). **b** Heatmap of mock- and Fg-treated hydrolysis samples. **c** Box plots of feature areas of the green (C1), red (C2) and yellow (C3) feature cluster in the heatmap in **b**

In addition to the technical characterization of the presented workflow for analysing soluble and cell wall-bound metabolites, a biology-oriented proof-of-concept study was also carried out. Many Phe-derived plant metabolites are known to be involved in plant defence and some of them are presumably capable of mediating resistance against (a)biotic stress by acting as building blocks for cell wall fortification [17, 35–37]. Conversely, the intermediates of putative building blocks may be released by hydrolysis and subsequently be present in the resulting cell wall hydrolysates. In the proof-of-concept study, water-treated control wheatear samples (mock treatment) were compared with *Fusarium graminearum*-treated wheatears (Fg treatment). Cultivation, treatment, and incubation were carried out according to established protocols similar to experiments described earlier [38]. The following comparison is purely based on the hydrolysates obtained from the two treatments. The scores plot after principal component analysis (PCA) is based on the Phe-derived metabolites (Fig. 6a) and shows a clear separation of the Fg and mock samples. Together with the heatmap obtained from two-dimensional hierarchical cluster analysis (Fig. 6b), unsupervised multivariate statistics revealed that metabolite profiles of hydrolytically released polyphenols clearly differed between Fg- and mock-treated samples.

Interestingly, the majority of the detected metabolite features showed increased abundance in the hydrolysate of Fg-treated compared to mock samples (red cluster C2, Fig. 6c). A second group of features showed, on average, similar abundance for both experimental conditions, which indicates that these metabolites were apparently not influenced by pathogen treatment (yellow cluster C3, Fig. 6c). It should be mentioned that potential differences might be obscured by the lower analytical precision observed for these features. This lower precision might partly be caused by the applied analytical conditions in general, or by low metabolite abundance. Moreover, a small group of features was more abundant in the mock compared with the Fg-treated samples (green cluster C1, Fig. 6c).

Interestingly, coumaric acid, ferulic acid, pinoresinol tricin and vanillin, which were already shown above to be most abundant in the hydrolysates compared to the extracts (Fig. 5), are all assigned to the red cluster. As these metabolites also showed higher abundance in the Fg-treated hydrolysate samples, these results suggest that they may contribute to plant defence by being incorporated into the cell polymer matrix. In addition, numerous features were annotated as di- or triferulic acid conjugates, similar to the findings of Bento-Silva et al. [10]. In summary, our results suggest the increased incorporation of Phe-derived metabolites into the cell wall in the presence of *Fusarium graminearum*. An in-depth biological interpretation of these metabolites is beyond the scope of this manuscript and will be presented elsewhere.

## Conclusions

A stable isotope-assisted workflow in combination with cell wall hydrolysis has been developed, which allows the profiling of both soluble and bound polyphenols in plant samples of interest. Exhaustive extraction of soluble metabolites prior to alkaline cell wall hydrolysis was achieved by three consecutive extractions. The presented method allows us to extend plant metabolomics studies to cell wall-bound phenylpropanoids after the application of ^13^C-Phe as a stable isotope tracer to metabolically active plants and thereby enables us to track descendants of polyphenolic cell wall constituents in an untargeted yet guided manner. This was confirmed by detection of expected hydrolysation products following the quantitative extraction of soluble metabolites. The protocol can be used in typical untargeted metabolomics studies. This is confirmed by the presented proof-of-concept study on pathogen-treated wheatears where numerous Phe-derived metabolites were differently abundant in cell wall hydrolysis samples of the two tested experimental conditions. Since changes in soluble polyphenols, as well as alteration of cell wall composition and/or cell wall architecture, can be expected to occur under various biotic and abiotic stress scenarios, the presented approach can help to enhance current plant metabolomics studies. The combination of the two approaches, namely stable isotopic labelling and cell wall hydrolysis, will allow a more comprehensive picture of phenylpropanoids and their contribution to plant defence or resistance with a special focus on those Phe derivatives that are not easily accessible by standard liquid extraction methods.

## Supplementary information

ESM 1 (XLSX 11.7 kb)

ESM 2 (XLSX 732 kb)

ESM 3 (XLSX 12.5 kb)
